# Empathy in patients with schizophrenia and antisocial personality disorder

**DOI:** 10.1192/j.eurpsy.2023.920

**Published:** 2023-07-19

**Authors:** K. Tasios, A. Douzenis, R. Gournellis, I. Michopoulos

**Affiliations:** 1 2nd Department of Psychiatry, Medical School, “Attikon” Hospital, National and Kapodistrian University of Athens, Athens, Greece

## Abstract

**Introduction:**

Violent behavior has been linked to deficits in social cognition, namely cognitive and affective aspects of empathy. Schizophrenia and antisocial personality disorder have been associated with violence and empathy deficits.

**Objectives:**

Our main objective is to search for differences in empathy between patients with schizophrenia who have committed a violent offence, patients with schizophrenia with no history of violent offence and patients with antisocial personality disorder.

**Methods:**

A total sample of Ν=100 participants was divided into four groups: 1) 27 patients with schizophrenia and history of committing a violent offence, 2) 23 patients with schizophrenia with no history of committing a violent offence, 3) 25 participants with antisocial personality disorder and 4) 25 general population participants comprising the control group. Symptoms of schizophrenia were rated using the Positive(P), Negative(N) and General Psychopathology (G) subscales of the Positive and Negative Syndrome Scale (PANSS). Empathy was evaluated using a) The Empathy Quotient (EQ). Theory Of Mind was evaluated using a) The First Order False Belief task, b) The Hinting task, c) The Faux pas Recognition Test and d) The Reading the Mind in the Eyes Test (Revised).

**Results:**

The four groups differed in PANSS scoring (p<0.001), EQ scoring (p<0.001) and Theory of Mind tests (p<0.001), but this difference was only significant between the controls and the three groups of patients. The three groups of patients did not differ to each other in any of the Theory of Mind tests. No difference was also found between the two groups of psychotic patients.

**Conclusions:**

Patients with antisocial personality disorder, schizophrenia and schizophrenia with a history of violent offence do not seem to perform differently in affective and cognitive empathy tests.

**Disclosure of Interest:**

None Declared

